# What is the IPCC’s assessment style and what shaped it?

**DOI:** 10.12688/openreseurope.18095.1

**Published:** 2024-08-07

**Authors:** Gabriel Târziu

**Affiliations:** 1 Munich Center for Mathematical Philosophy, LMU Munich, Munich, 80539, Germany

**Keywords:** the IPCC, IPCCs objective, criticism of IPCC, IPCCs assessment process

## Abstract

The Intergovernmental Panel on Climate Change (IPCC) is one of the best-known global expert organizations. Its main objective is to supply policymakers with policy-relevant recent scientific information about climate change. The way in which the IPCC is obtaining this information is called an assessment. But assessments can be performed in a wide variety of ways. An important step, then, in understanding what this important organization does and why, is to figure out what characterizes the particular type of assessment it performs. The main objective of this paper is to contribute to the literature dedicated to understanding the work of IPCC by outlining the characteristics of the IPCC's assessment style and providing an in-depth analysis of the factors that have contributed to its development. As it will be argued here, understanding the climate-scientific-specific obstacles that had to be overcome by the IPCC in the process of pursuing its objectives is crucial for understanding why the IPCC is performing the type of assessment that it does and also for understanding some of the most important controversies associated with it.

## 1. Introduction

The Intergovernmental Panel on Climate Change (henceforth IPCC or the Panel) is a crucial organization in humanity’s struggle to deal with the problem of climate change. Part of its job is to provide policymakers with the necessary information for making the best decisions in addressing this problem. In its role as a science-policy mediator, the Panel underpinned important multilateral environmental agreements on tackling climate change such as the Kyoto Protocol and the Paris Agreement. But what exactly is it that the Panel is doing, why is it doing it in that particular way and why what it does was/is perceived by some as controversial? 

We can distinguish between two different strategies for tackling these questions. The first strategy is to focus on the social and political factors that played a crucial role in the setting up of the IPCC and that influenced its subsequent development. This is the most widespread approach found in the literature on the IPCC. A different strategy is to try to answer these questions by concentrating on the climate-scientific-specific obstacles that the Panel had to overcome in trying to do what it has been mandated by the UN General Assembly (UNGA) to do, and on how the solutions the Panel found for dealing with these obstacles shaped its assessment style and also generated controversies.

Both these approaches have their merits. The first one is better at accounting for the procedural ^1^ characteristics (i.e. those aspects that concern the structure of the organization, how the assessment ^2^ process takes place, who is involved in it and why, and how the assessment reports get evaluated) of the IPCC while the second one can be perceived as being better at explaining the substantive aspect (i.e. those aspects that concern the type of assessment performed in terms of level of research, its scope, and the detail and integration of the information presented) of the assessment performed by the Panel. Of course, the two strategies are complementary and can be used together to provide a comprehensive answer to the questions asked above about the IPCC.

This paper is meant as an exercise in the second type of strategy. As such, it should not be confused with a historical account, a social scientific explanation, or a review of the literature on some specific aspect of the IPCC. What follows is the result of an epistemological effort to identify those climate-scientific-specific factors that were at least very important, if not crucial, in shaping the IPCC’s assessment style. The first step in this effort, to which Section 3 is dedicated, will be to make clear the fact that there are indeed different assessment styles, i.e., that assessments can be performed in a variety of ways. A second step ( Section 3.1. and Section 3.2.) is to argue that the drive to satisfy the needs that engender it and what World Meteorological Organization (WMO) and the United Nations Environment Programme (UNEP) tasked it to do were insufficient to force the Panel to adopt a particular type of assessment. Section 4 will be dedicated to figuring out what exactly is IPCC’s own perception of its assessment style. In Section 5, the objective is to argue that a series of climate-scientific-specific obstacles that made pursuing the IPCC’s objectives a very hard job was critical in shaping its assessment style. Section 5.1. will list five important aspects of climate research that can be perceived as obstacles that stood in the IPCC’s path to a straightforward achievement of its objectives and will try to make the connection between the IPCC’s solutions to overcoming these obstacles and the type of assessment the IPCC ended up performing. And, finally, Section 5.2. will discuss some of the controversies associated with the Panel’s assessments and show that they have to do with the particular way in which the IPCC dealt with the obstacles. The starting point and the stage for the entire discussion will be set up in the next section.

## 2. Why did we need an IPCC to begin with? A preliminary, simple story

The IPCC was established in 1988 by the WMO and the UNEP. One story that can be told about the genesis of this organization may take the need for it as being kindled by two important developments in the 1970s and 1980s. ^3^ The first one has to do with a series of global atmospheric/climatic issues that occurred in that period, such as the acid rain caused by the lowering of the pH of rainwater by the sulphur dioxide from the burning coal that drifted into the atmosphere, the discovery in 1985 of a hole in the ozone layer over Antarctica after years of warnings issued by scientists about the fact that man-made chemicals were harming the ozone layer, the great Sahelian drought of the 70s and 80s that lead to hundreds of thousands of people dying from starvation, and the 1972 European drought that lead to the Soviet crop failures in that period and the ‘Great Grain Robbery’ wheat deal of 1973 between the United States and the Soviet Union that caused a global food price increase by at least 30 percent. All these, together with the growing concern in that period about the effects that a potential nuclear war could have on Earth’s climate (i.e. the nuclear winter scenario), made clear two things for the world’s governments: that human activities can not only cause local or regional pollution-related problems but have the potential to negatively impact the entire planet by affecting the Earth’s atmosphere, and that climatic changes can have serious economic consequences and can lead to the loss of countless human lives. The interest to know more about the extent of this impact and to quantify the human influence on climate led to the creation of national (e.g. the United States Climate Program) and international (e.g. the World Climate Research Programme) organizations aimed at climate monitoring, climate forecasting, and basic research, and so it boosted the financing of climate research.

 The result was a significant increase in climate research output all over the world. In this context, the need for establishing an IPCC was first suggested, eight years before its creation, by Aksel Wiin-Nielsen, the WMO Secretary-General between 1980 and 1983.

According to Wiin-Nielsen,

“there is a real need for some machinery to maintain regular critical scientific appraisal of... the research in a form which also renders possible definitive and authoritative statements from time to time interpreting the results in terms meaningful to those responsible for policy... These requirements could best be met by some form of international board for the assessment of all scientific aspects of the CO2 question” (A. Wiin-Nielsen quoted in Edwards, 2010, p. 377).

As it transpires from Wiin-Nielsen's remark, in the 80s, governments and climate scientists were starting to feel the need for an organization that could do at least the following two important things: keep track of the fast-growing climate research conducted all over the world and compile a list of findings that could be communicated to the policymakers in a way that was meaningful and useful to them. So, the IPCC's main *raison d'être* had to do with fulfilling these two tasks which are coded in the first two objectives that WMO and UNEP set up for the IPCC:

“(i) to make assessments of available scientific information on climate change;
(ii) to make assessments of environmental and socio-economic impacts of climate change;
(iii) to formulate response strategies to meet the challenge of climate change” ( United Nations General Assembly, 1988).

The third objective was meant to help with the policy process even more in a way that was not policy prescriptive, by providing the policymakers with clear information about the potential implications that different response strategies would end up having in terms of climatic and societal impacts.

## 3. Different types of assessment and possible constraints

Everything may seem clear, uncomplicated, and uncontroversial until this point: some climate-related developments in the 1970s and 1980s that were linked (in the opinion of many scientists at that time) to human activities triggered the interest of the world’s governments to know more in order to take, if possible, the required actions to prevent economic harm and loss of human lives. This (together with a number of convoluted social and political factors) ^4^ lead to the establishment of the IPCC. But what exactly is it that the IPCC is doing? This also may be perceived as an easy question. After all, only a glimpse at the literature on the IPCC may seem to provide us with more than we need for an answer: the IPCC is a global expert organization that supplies policymakers with policy-relevant recent scientific information about climate change. The way in which the IPCC is obtaining this information is called an assessment. ‘Assessment’ is usually taken in the literature to refer to the process by which expert knowledge related to those problems that are of special interest to society is generated and used to inform policy-making (e.g. Castree *et al.*, 2021; Jabbour & Flachsland, 2017; Oppenheimer *et al.*, 2019).

 Of course, the above answer to the question about what it is that the Panel is doing is not mistaken. The problem with it is that it can only get us so far in understanding the IPCC. We realize this the moment we take into account the fact that there is no single way in which assessments can be performed. As discussed in Castree, Bellamy, and Osaka (2021), a great number of assessments have been done since the late 70s (more than 140) and although there are some things that they all have in common, the difference between them is so significant that, about the majority of them, we can only claim that they bear a family resemblance (p. 57). One reason for this is that there are many different ways in which we can deal with a problem. One can, for instance:

- Use the existing knowledge to determine whether the problem is real or not;
- Produce new knowledge relevant to the particular problem;
- Use the existing knowledge to find solutions to the problem;
- Keep track of the work done in science that is relevant to the problem;
- Identify gaps in the scientific knowledge relevant to the problem and, by doing this, establish research priorities and stimulate research that can be used to solve the problem;
- Try to quantify the uncertainty of the scientific findings that are relevant to the problem;
- Provide expert opinion on problems that involve a high degree of uncertainty or about which there is a lack of sufficient information for a proper scientific quantification.

All these different ways of dealing with a problem or a combination of them can be used to produce different types of assessments. However, this is not the only way in which assessments can vary. Parson *et al.* (1997), for instance, claim that assessments can also “show great variation in the questions they address, the procedures by which they operate, and the substance of the outputs they deliver” (p. 53). According to Parson and his collaborators, one way to distinguish between variations across assessments is in terms of the following substantive characteristics: scope, detail, integration, novelty and level of research, and policy specificity (p. 54). Take, for instance, the novelty and level of research aspect. One way in which an assessment may proceed with respect to this aspect is by being as faithful to the existing research as possible and so by producing only a survey of the existing knowledge on the topic of interest. A completely different approach is to undertake and present new research pertaining to the problem of interest. Some assessments have been undertaken along the lines of the first approach (e.g. the 1982 report edited by William C. Clark on the CO2 buildup in the atmosphere), some along the lines of the second (e.g. the Scientific Committee on Problems of the Environment presented new research in its 1986 report), while others preferred a combination of the two (e.g. the National Acid Precipitation Assessment Program combined new research with a survey of existing knowledge in its 1991 report).

I think enough has been said to understand, by now, why saying about the IPCC that it is a global expert organization in the business of assessing that information relevant to the issue of climate change doesn’t provide us with a complete picture of what the Panel is doing. For a more substantive image, we also need to answer the following question: what type of assessment is the IPCC performing and why? This question can be tackled from at least two different directions. The first one is by looking at what the UNGA mandated the IPCC to do. A different path that one may prefer for answering our question is to think about the Panel in terms of the needs that it was meant to satisfy and the best way of satisfying those needs. The aim of the following two sections ( 3.1. and 3.2.) is to show that neither the mandate of the WMO and the UNEP nor the drive to satisfy the needs that engendered it could have counted as strong constraining factors for the particular assessment style that the IPCC ended up adopting.

### 3.1. Is the IPCC’s assessment style constrained by what the WMO and UNEP tasked it to do?

One direction in which one may look to figure out what assessment style is characteristic of the IPCC and why is the resolutions by the UNGA, WMO, and the UNEP for its establishment. What one can find out by looking in that direction is this: “Following the mandate in the UN General Assembly Resolution 43/53 of 6 December 1988 ( UNGA, 1988), the IPCC was designed to serve global policy with scientific information on the nature, impacts and policy implications of climate change” ( Livingston *et al.*, 2018, p. 83). As it has been discussed above ( Section 2), the WMO and the UNEP decided that the IPCC should pursue three main objectives. But in their resolutions, none of IPCC’s parent organizations mentioned anything about the type of assessment that the IPCC was meant to perform. This led to the following situation: the leaders of the IPCC didn’t have a very clear idea in the beginning about the way the Panel had to pursue its objectives. So, they had to create their own rules and decide on the appropriate assessment style. As Bert Bolin (the founding chairman of the IPCC) recounts in his overview of the history of IPCC:

“It was clear to the leaders of the IPCC that we had to develop our own procedure for how to achieve the task that had been given us. During the first couple of years we formally followed the WMO procedures when in doubt. This lack of more precise rules of procedure for a task that was going to be rather different from the ordinary WMO activities gave the IPCC great flexibility in handling matters and could be exploited to the advantage of the assessment process, but care had to be exercised. It gradually became apparent, however, that we had to become more strict and professional in our work...” ( Bolin, 2007, pp. 5–51).

By its fifth meeting (March 1991) IPCC did manage to come up with its own set of rules ( Bolin, 2007, p. 70) which were subsequently amended several times over the years ( IPCC, 2018).

### 3.2. Is the IPCC’s assessment style constrained by the needs that engendered it?

The previous section showed that the way the IPCC was mandated by its parent organizations left a lot of freedom concerning the way its objectives were to be pursued and so with regard to the type of assessment that the Panel was supposed to perform. Did the need that engendered the IPCC act as a more important constraining factor on its assessment style? Not really, no. To understand why this is so, let’s consider the following question: what need(s) are we referring to? We may use the discussion in Section 2 to provide the following answer: the need to keep track of those scientific findings relevant to the issue of climate change and keep the policymakers informed about important developments. But is this the whole story about the relevant needs that can be associated with the establishment of the IPCC? Of course not. This only scratches the surface. We can also speak about political needs, as is being done, for example, in Agrawala (1998a). Another type of need that is unquestionably relevant in this context is the policymaking needs, i.e. those needs that appear in the context of designing and implementing climate change-related policies. Yet another highly relevant type of need is that having to do with environmental protection. The WMO, in its decision about the setting up of the IPCC, takes as relevant the following research-related set of needs:

“BEING AWARE OF:
  2. a need to
  (a) maintain and develop further an efficient long-term monitoring system, making it possible to diagnose accurately the current state of the climate system, the trends, and the factors having an influence on climate,
  (b) improve our knowledge of the sources and sinks of the major radiatively important trace gases (‘greenhouse gases’), and develop more reliable methods for predicting their future atmospheric concentrations,
  (c) promote research aimed at closing the gaps in our ability to understand and predict the climate system, including reliable projections of the regional distribution of the expected climate change.
AGREES:
  that an IPCC should be established.” (quoted in Bolin 2007, pp. 51–52)

What this should make clear is that, when it comes to the needs that were important in setting up the IPCC, one may tell a variety of different stories depending on the preferred perspective. This raises the following question: was it the political needs, the socio-economic needs, the environmental protection needs, the policymaking needs, or the research-related needs that played a crucial part in shaping the IPCC’s assessment style? I believe that the correct answer to this question is “neither one of the above” and this is for two important reasons. First of all, a need may be satisfied in a variety of ways. Take, for instance, the research-related needs pertaining to a particular problem. Each one of the six different ways of dealing with a problem discussed above can be perceived as a good way of satisfying the need. Second of all, even if one may be able to make a strong argument for why a particular type of needs played the most significant part in IPCC’s genesis that doesn’t exclude the possibility of other needs being relevant as well. But different needs may pull in different directions and so they end up underdetermining the choice of a particular assessment style.

So, to wrap things up, what the discussion in this section was meant to show is that “an assessment’s context cannot fully determine how it is done” ( Parson *et al.*, 1997, p. 55) and this is for two important reasons: (a) it is very difficult to determine which needs were more important and why, and (b) a strong argument may be made for the underdetermination of the assessment styles by the set of needs associated with it.

## 4. What characterises the type of assessment that the panel is performing?

Let us remember that the main focus of this paper is on the characteristics of the type of assessment that the IPCC is performing and the main question that is addressed here is: why exactly did the IPCC adopt that particular assessment style, and why is it perceived by some as controversial? What has been done in the previous sections was to show that a satisfactory answer to this question cannot be obtained by looking at the IPCC’s objectives (i.e. at what it was mandated by the WMO and UNEP to do) or at the needs that engendered it. So, if we are to understand what the IPCC is doing, i.e. the special assessment style that characterises its activity, we have to look in a different direction for an answer to these questions. But before getting to that part of the discussion, we need to fill an important gap: saying a few words about what it is that characterises the type of assessment performed by the IPCC. This section will be dedicated to filling this gap and the preferred method for doing this will be to look at what the IPCC is saying in its last assessment report (AR6) about what it does. As, I hope, this section will make clear, an important characteristic of IPCC’s assessment style is that it embodies conflicting tendencies ( Oppenheimer *et al.*, 2019, p. 2005).

So, what exactly is the IPCC saying it is doing? If we browse only through IPCC's Working Group I (WGI) latest report we find a lot of different answers to this question. Here are a few examples:

1. [IPCC assesses the] “likelihood of an outcome or a result” ( IPCC, 2021, p. 38)
2. [IPCC provides an] “overall assessment of Earth’s sensitivity to climate forcing” ( IPCC, 2021, p. 45)
3. “In all three Working Groups, author teams evaluate underlying scientific understanding and use two metrics to communicate the degree of certainty in key findings” ( IPCC, 2021, p. 169)
4. “Working Group I (WGI)... assesses the current evidence on the physical science of climate change, evaluating knowledge” ( IPCC, 2021, p. 150)
5. [The IPCC is concerned with providing] “a comprehensive assessment of the scientific literature” ( IPCC, 2021, p. 153)
6. “The assessment covers scientific literature accepted for publication by 31 January 2021” ( IPCC, 2021, p. 40, footnote 8)

A closer look at these quotes reveals that WGI is using at least three different meanings of ‘assessment’ ^5^ in its report. The first meaning is evident in the first two statements. In these cases, ‘assessment’ is tantamount to performing the tasks of quantifying the uncertainty in key findings and of providing answers to scientific questions (such as “What is the value of ECS?”). In the next two claims (3 and 4), ‘assessment’ means getting involved in the epistemological enterprise of evaluating the cognitive achievements made in climate science. In the last two quotes (5 and 6), ‘assessment’ means the task of conducting a systematic literature survey to synthesize the research to enhance the “visibility of key knowledge developments that are potentially relevant for policymakers” ( IPCC, 2021, p. 154).

Unfortunately, this ambiguity in what is meant by ‘assessment’ creates a lot of confusion about what it is that the IPCC is doing and about how exactly to interpret the claims that it makes in its reports. For instance, when it claims that:

(A): “The AR6 assessed best estimate [for ECS] is 3°C” ( IPCC, 2021, p. 11).

what exactly is the Panel saying? We can give different answers to this question, depending on what we take ‘assessment’ to mean in this context. For instance, we can say that what we are dealing with is a scientific claim, i.e. that this estimate for ECS is the result of IPCC’s research into the problem of the value of ECS. Alternatively, we can take (A) to be the result of a thorough survey of the scientific literature. Finally, we can understand (A) as saying that climate scientists have a good grasp of this value because they satisfy the epistemological criteria for understanding, e.g. they have a good explanation for why 3°C and not some other value should be taken as the best estimate for ECS.

Taking a closer look at what the Panel is doing in the assessment reports is of no help when it comes to clarifying this issue. I will try to substantiate this next.

According to one of IPCC’s unwritten rules, the Panel does not conduct its own research ( IAC, 2010; Yohe & Oppenheimer, 2011, p. 633. See also the claims regarding this made by the Panel on https://www.ipcc.ch/). Then, statement (A) can be taken as the result of IPCC’s survey of the scientific literature on the value of ECS. This means that we should interpret (A) as saying that this estimate (i.e. 3°C) for ECS is a key knowledge development in climate science that the IPCC uncovered through a systematic literature review.

Some of the claims made in AR6 support a different interpretation, though. In Chapter 7 it is claimed, for instance, that “New approaches to the quantification and treatment of feedbacks (Section 7.4) have *improved the understanding* of their nature and time-evolution, *leading to a better understanding* of how these feedbacks relate to equilibrium climate sensitivity” ( IPCC, 2021, Ch.7.1, p. 929, my emphasis). All these references to “understanding” make it sound as if the Panel is involved in the epistemological enterprise of assessing the degree of scientific understanding of ECS and the advances in understanding made over time in climate science regarding the exact value of ECS. This interpretation gets a lot of support, also, from what is said in the description of the energy budget framework presented in Box 7.1: “The framework reflects *advances in the understanding* of the Earth system response to climate forcing since the publication of AR5” ( IPCC, 2021, Ch.7.1, p. 931, my emphasis).

Getting deeper into the details of IPCC’s discussion of this topic reveals yet another possibility. Although the Panel claims it doesn’t perform research, the way it comes up with the best estimate for ECS suggests otherwise. Some of the decisions WGI made in coming up with the value of 3°C for ECS, such as applying “weights to each model before averaging them, to produce ‘assessed global warming’ projections” ( Hausfather *et al.*, 2022, p. 28) ^6^ and excluding direct estimates of ECS based on earth system models ( IPCC, 2021, p. 1007), ^7^ go beyond the climate scientific literature on this topic. This is something that the Panel seems to acknowledge in the following statement: “this Report’s assessment is largely based on observations and an improved understanding of the climate system” ( IPCC, 2021, FAQ 7.3, p. 1024). So, IPCC’s best estimate for ECS is not extracted from the literature through systematic review but is based on the report’s authors’ scientific expertise.

## 5. How did we get here? 

Probably, the best way to summarize the discussion in the previous section is with the following quote from Oppenheimer *et al.* (2019): “In sum, the IPCC embodies conflicting tendencies and inclinations” (205). But why? How did we get here? The rest of this paper will be dedicated to showing that the reason for this has to do with the fact that, even though apparently simple, the objectives that WMO and UNEP set up for the IPCC proved to be incredibly hard to pursue. What made them so was a series of climate-scientific-specific obstacles. Trying to overcome these obstacles had a powerful impact on the Panel’s assessment style and generated some of the most important controversies associated with the Panel. As the next section will try to make clear, dealing with these obstacles forced the IPCC to shape its assessments in such a way as to include consensus-generating, uncertainty-quantification, quality-checking, hole-filling, and evidence-gathering activities.

### 5.1. Obstacles and assessment characteristics

The first obstacle the made the IPCC’s job a very hard one concerned the fact that a lot of climate research was being undertaken by governmental organizations and other non-academic institutions and their research output didn’t always undergo the normal scientific scrutiny (in the form of e.g. peer review) which means that they could contain hidden errors. A well-known example is the one that captured the attention of the media in early 2010: the case of the melting of the Himalayan glaciers. A report published in 1999 by the Working Group on Himalayan Glaciology (WGHG) of the International Commission for Snow and Ice (ICSI), a body of the World Wildlife Fund (WWF), an environmental organization, stated that Himalayan glaciers could disappear by 2035. But, as many scientists around the world made clear (and as the authors of the report acknowledged ( WWF, 2005, p. i)), this is highly unlikely to happen even if the thinning rate of the glaciers increases dramatically compared to recent trends ( Kargel *et al.*, 2011). What episodes similar to this one made clear was that the IPCC could not just uncritically incorporate in its reports all the climate-scientific research results but had to verify them beforehand, i.e. it had to perform a quality check of the literature.

 A second obstacle had to do with the lack of consensus in the climate scientific literature on key findings. Take for instance the case of the equilibrium climate sensitivity (ECS). ECS is a metric used to quantify how much warming is generated (by the time the climate has reached a new equilibrium) by a doubling of the CO2 in the atmosphere relative to the pre-industrial climate. ECS is probably the most important metric for IPCC because it is crucial for determining how serious the anthropogenic impact on climate is. Unfortunately, the climate-scientific community is highly divided when it comes to the value of ECS. For example, in its fifth assessment report (AR5), IPCC estimates that ECS is *likely* in the 1.5 – 4.5 range, but climate models-based estimates and estimates that rely on paleoclimate records appeared (at the time of AR5) to rule out ECS values in the lower range (i.e., ECS smaller than 2). For IPCC to come up with the 1.5 – 4.5 range, it had to create or force consensus on this issue by convincing the climate scientists engaged in modelling about the relevance of several studies that derived ECS from the instrumental records of historical temperatures and that calculated a lower range for ECS. This obstacle influenced the IPCC’s particular assessment style by forcing the Panel to act as a consensus-generating machinery (of course, as we will see below, this characteristic of the IPCC’s assessment style led to controversy because the consensus that it generates has been perceived as artificial, detrimental to scientific practice, and not at all representative of the diversity of views found in the literature).

 Yet another problem that affected the type of assessment the IPCC is performing had to do with uncertainty. Even if there would be widespread agreement in the scientific community on a specific range for the ECS, for instance, this range would still not constitute a *definitive statement* (see the quote from Wiin-Nielsen in Section 2) about ECS because there is still a lot we don’t know about Earth’s climate system and so all the climate-scientific results are (to a certain degree) uncertain. This, of course, is less than optimal if the aim is to provide assessments that are meant to be policy-relevant. One way to deal with this problem is to try to quantify the uncertainties. But what is the best way to characterize and quantify the climate-scientific uncertainties? This is a question that the Panel had to address. Since its inception, the management and reporting of uncertainties have been a big topic on IPCC’s agenda ( Mastrandrea & Mach, 2011). Unfortunately, the first assessment reports didn't use a consistent framework for communicating uncertainties across the three Working Groups. Such a framework was created by the IPCC starting with its third AR and has constantly been improved since then ( Mach *et al.*, 2017; Mastrandrea *et al.*, 2010; Mastrandrea *et al.*, 2011; Moss & Schneider, 2000).

 A fourth obstacle that influenced the IPCC’s assessment style has to do with the fact that there is no single scientific discipline responsible for generating knowledge relevant to what Wiin-Nielsen called “the CO2 question.” There are, in fact, dozens of scientific disciplines (e.g., Climatology, Oceanography, Glaciology, Paleoclimatology, Ecology, Human geography, Economics, etc.) that together generate this type of knowledge. This situation forced the Panel to act as an interdisciplinary knowledge integration system. That it indeed assumed this function is clear from the following statement from the preface to its latest AR: “The report recognizes the interactions of climate, ecosystems and biodiversity, and human societies, and integrates knowledge more strongly across the natural, ecological, social and economic sciences than earlier IPCC assessments” ( IPCC, 2021, WG2, p. ix).

 Of course, the problem with the knowledge relevant to the CO2 question is not only that it is scattered across different disciplines, it is also that there are gaps in it. This constituted a fifth important factor that constrained IPCC’s assessment style: it couldn’t just provide a synthesis of knowledge but had, in some cases, to step in and fill important knowledge gaps ( Tol, 2011, Yohe & Oppenheimer, 2011). For example, in AR4, the IPCC took it upon itself to provide an estimate of the contribution that the large ice sheets (Greenland and Antarctica) may have to future sea level rise ( Oppenheimer *et al.*, 2007). A second, and arguably more important, knowledge gap that the IPCC tried to fill concerns the future anthropogenic greenhouse gas emission scenarios ( Yohe & Oppenheimer, 2011). As noted by Bolin, at the time of the first IPCC report, “the construction of emissions scenarios for greenhouse gases had not yet attracted much attention from the scientific community” ( Bolin, 2007, p. 65). IPCC’s Working Group III took upon itself the task of developing a set of four such scenarios. Given the fact that the most important source of uncertainty affecting global climate projections is represented by our lack of knowledge about future anthropogenic emissions, this is quite an important contribution. Another important knowledge gap that the IPCC tackled by generating its genuine scientific output concerns the topic of abrupt changes and tipping points. One way in which the Panel addressed this problem was via expert judgements, but, as argued by Lam and Majszak (2022), “expert judgment about climate and Earth system tipping points is actually often used as a genuine scientific output, complementary to – but not independent of – model outputs” (p. 9).

### 5.2. Obstacles and controversies

The previous section was dedicated to showing that the type of assessment performed by the IPCC was strongly influenced by a series of climate-scientific-specific obstacles. As a result of trying to overcome these obstacles, the Panel ended up being involved in several activities (such as quality-checking, uncertainty-quantification, holes-filling, and evidence-gathering) that determined its particular assessment style. This section aims to show that dealing with these obstacles is also the source of some of the most important controversies associated with IPCC’s activities.

In trying to overcome the first obstacle (i.e. the hidden errors in the research), the IPCC had to act as an epistemic authority in charge of providing quality control for all the research output relevant to the question of climate change. However, there are several problems associated with IPCC assuming such a position. One of these has to do with satisfying the criteria for it: does the IPCC have what it takes to be considered an epistemic authority on climate change? This is not an easy question to answer because it is not clear what is needed to claim such authority in the first place. What I believe is uncontroversial is that putting together (to achieve the objectives laid out by WMO and UNEP) a bunch of scientists nominated by the governments of member countries, and whose activities and reports are politically supervised, clearly isn’t enough for it. But it is not at all obvious that the IPCC does more than that.

Also, even though it managed to mobilize a lot of scientists from all over the world, the Panel still lacks the resources to eliminate all the errors hidden in the literature and so some mistakes can still sneak, through its assessment process, into its reports. This is exactly what happened in IPCC’s AR4 with the case of the melting of the Himalayan glaciers. As we saw above, a report by WGHG stated mistakenly that Himalayan glaciers could disappear by 2035. As a scientific authority on all things pertaining to climate change, the IPCC should have spotted this mistake. But it didn’t. So IPCC’s Working Group II (WGII) ended up including it in its report ( Anderegg *et al.*, 2014, p. 1448). According to Kargel *et al.* (2011),

“this error... shredded the reputation of a large and usually rigorous international virtual institution. The gaffe by the Intergovernmental Panel on Climate Change helped to trigger a global political retreat from climate change negotiations, and it may prove to have been one of the more consequential scientific missteps in human history” (p. 14709).

This last statement is clearly an exaggeration of the impact that this episode had ^8^ on the scientific credibility of the IPCC and on the international community’s attitude towards the issues of fighting climate change since the IPCC continues to command substantial trust in the present day and tackling climate change remains very much a global policy priority. But it does bring to the forefront another problem associated with assuming the status of an epistemic authority. Having this status means that the IPCC is in the position of “speaking with one voice for global climate science" ( Hoppe & Rödder 2019). But this position doesn’t leave room for mistakes because any mistake affects the credibility of the entire climate-scientific community and it has the potential to negatively impact the policy-making process.

A good thing about IPCC’s epistemic authority status is that, by occupying this position, it has the power to force scientific consensus ^9^ on key climate scientific results. ^10^ According to Boers, Ghil & Stocker (2022):

“The preparation of an IPCC assessment report is essentially a consensus-building process. It forces the scientific community to assess the current state of knowledge on a specific issue, formulate a consensus where possible, map out the uncertainties...” (p. 17).

This is good because it addresses the second problem discussed in Section 5.1. (i.e. the lack of consensus on key findings), but, most importantly, because, according to some authors, ^11^ it plays a crucial role in convincing the policymakers (and the general public) that human-caused climate change is real and so it is essential for generating greater support for public action. Unfortunately, it is also an important source of controversies.

According to Yohe & Oppenheimer (2011), “the emphasis on consensus is the most troublesome limitation of IPCC assessment processes” (p. 634). This limitation has repercussions both on policymaking and on the climate-scientific practice. One way in which the emphasis on consensus may end up hurting policymaking is by ignoring or downplaying extreme possibilities ( Oppenheimer *et al.*, 2007). By being the product of a consensus approach, the IPCC reports inadvertently end up hiding important information from the policymakers and so manage to shield them from understanding more extreme possibilities which, potentially, could be important for taking more nuanced climate change mitigation actions. Other authors identify other important problems with IPCC’s emphasis on consensus. For instance, IPCC’s approach is taken by Curry (2011) to end up masking the complexities of the climate change problem and the associated uncertainties in our understanding, and by Van der Sluijs *et al.* (2010) to underexpose matters over which there is no consensus. According to Hulme (2022), it also encourages premature agreement among experts where there is none and may come across as unfair and unaccommodating to the full range of accredited views.

Besides standing in the way of good policymaking, the consensus approach can have additional negative effects by interfering with the normal climate scientific practice. One significant concern is that in emphasizing consensus, the underlying scientific foundations of individual studies can be overlooked and so this approach may lead to a loss of appreciation for the merits and nuances of each study, and, more importantly, to a failure to identify avenues for making further progress ( Stevens *et al.*, 2016, p. 514).

The next obstacle that was approached by the IPCC in a way that generated controversies concerns the treatment of uncertainty. Given the characteristics of the climate system, the evolving nature of its research, the difficulties in anticipating what we as a society will do in the future, etc., the information about climate change has inherently uncertain components and so cannot be expressed as statements of facts. What this means is that, in its reports, to properly inform policymaking, the Panel has to provide a clear and coherent characterization of uncertainties and the confidence levels associated with the key findings. So, as stated by Oppenheimer *et al.* (2016), “Managing the risks of climate change requires a consistent and comprehensive approach to quantifying uncertainty and a clear narrative to describe the process” (p. 445). Has the IPCC managed to do that? No, it did not. I am not going to enter into details here about the IPCC’s failures in this regard. There is a lot of literature on this topic and surveying it would require a lot of space. ^12^ What I should say, though, is that IPCC's failure to create a good framework for the treatment of uncertainties is very unfortunate given the fact that the issue of uncertainty is a notorious weakness of the reports which has been effectively used by the climate change sceptics to argue against the necessity of taking any kind of climate actions.

The last two obstacles, i.e., putting together different lines of evidence and filling holes in research, got the IPCC involved in conducting new research. This means that it had to violate one of the most important unwritten rules regulating its activity: “IPCC shall perform no original research” ( Yohe & Oppenheimer, 2011, p. 633). This explains, at least partly, what is referred to above as the conflicting tendency that characterises IPCC’s assessment. The fact that the Panel decided to get involved in research was perceived by some as particularly troublesome. A strong case for why this is concerning is made, for instance, in Tol (2011). According to Tol, the Panel exerts a monopoly not only on climate policy advice but also on climate-scientific research. But, as it is well known, monopolies are usually associated with negative outcomes such as reduced competition, less innovation, and inferior products or services. According to Tol, all these downsides can also be associated with IPCC’s research: its outputs (such as emission scenarios) are more widely used than any of the alternatives, the Panel has not innovated much, and the quality of the assessment reports has declined over time.

## 6. Conclusion

The main objective of this paper was to explore a different strategy for accounting for an important aspect of IPCC’s activity: the type of assessment that it performs in order to obtain policy-relevant scientific information about climate change. Instead of focusing on the social and political factors that influenced its genesis, the discussion in this paper revolves around the climate-scientific-specific obstacles that the IPCC had to overcome in order to achieve its objectives. These obstacles include the need to address consensus-issues, the inherent uncertainty of climate-scientific results, the interdisciplinary nature of climate research, and the gaps in knowledge relevant to climate change. It is the contention of this paper that the Panel's response to these obstacles acted as both the main constraining factor on its assessment style and the principal source of controversies associated with it.

### Ethics and consent

Ethical approval and consent were not required.

## Data Availability

This paper does not involve the collection of empirical data, and so there are no specific datasets or materials associated with the study. All information and arguments presented in the paper are based on existing literature and the author's analysis. No data are associated with this article.
