# Supplementary material for: What is the IPCC’s assessment style and what shaped it?
Source: Open Res Eur. 2024 Aug 7;4:172. [Version 1] doi: 10.12688/openreseurope.18095.1 (PMC11494274; doi:10.12688/openreseurope.18095.1)
Supplement: Peer review history

# Reviewer report: Reviewer response for version 1

**DOI:** 10.21956/openreseurope.19558.r44599

**Published:** 2024-11-19

**Authors:** Ema Gusheva

**Affiliations:** 1 Delft University of Technology, Delft, The Netherlands

The article is a philosophical investigation of IPCC assessments on climate change. It aims to describe the  assessment in light of its goals/process and argument possible reasons why it is structured in that specific way. The scientific merit of this work could be in highlighting logical fallacies in the IPCC's aims and tasks, but a lot of improvement is needed. 

 First, the article would be improved by using more specific language instead of general terms and including more references for your claims. For example, you state that the way the IPCC goes about reaching its objective of supplying policymakers with policy-relevant scientific information is called an assessment. But what exactly is an assessment? Are all the tasks IPCC does part of the assessment? Are the IPCC communication efforts or the SPM approval process part of the assessment? It would be beneficial if you describe the IPCC assessment process, especially for readers less familiar with the IPCC. Moreover, given that your aim is to describe the IPCC assessment style, I recommend that you compare it in a more systematic way with comparable assessments. This can also be tied to your description of the IPCC assessment process. You discuss ‘climate-scientific-specific obstacles’? But how are you sure they are ‘climate-scientific-specific’ if you don’t compare them with other assessments? Similarly, you don’t explain or reference why "the IPCC assessment is perceived as controversial". There have been some controversies associated with the IPCC assessments but I am not sure the assessments themselves are controversial. Likewise, you state that “In its role as a science-policy mediator, the Panel underpinned important multilateral environmental agreements on tackling climate change such as the Kyoto Protocol and the Paris Agreement”. How did it underpin them? Can you provide some supporting references?

 Second, you cite some other works describing what an assessment is in footnote 2, but I think your work would benefit from a deeper look at the literature on scientific advice mechanisms for policy. For example, there is a difference between scientific research, scientific assessments and policy advice. In my view, assessments do not, as you claim, "Identify gaps in the scientific knowledge relevant to the problem and, by doing this, establish research priorities and stimulate research that can be used to solve the problem" and “Produce new knowledge relevant to the particular problem” (this is done by scientists in scientific research) or “Use the existing knowledge to find solutions to the problem” and “Provide expert opinion on problems that involve a high degree of uncertainty or about which there is a lack of sufficient information for a proper scientific quantification” (this is done by policy advisors in policy advice). If the IPCC merely assesses science, then any errors it produces would not be errors from the assessments but rather errors in the underlying science. Hence, it should not be held accountable for this, as you suggest in section 5. It also does not produce policy recommendations because its aim is to be policy-relevant not policy-prescriptive, thus it should not be held accountable if the policy ideas presented in the reports do not work in the real world. In fact, the only way in which the assessment can go wrong is if it is not comprehensive or if it misrepresents the underlying science.

 Third, for audiences unfamiliar with philosophical research paper, it would be good to clarify what what you mean by “this is the result of an epistemological effort” and why it is a good fit for the problems you raise.

 Fourth, one of the main arguments in the text is that, despite the IPCC claiming that its job is merely to assess and not conduct original research, some of the work report authors do can be understood as conducting research and that this is troublesome because it is not conducted systematically. However, the argument can be improved by clearly defining how 'assessment' and 'research' differ. It seems to me that your supporting example with ECS counters your argument because the reliance on the authors' scientific expertise is precisely how I would define 'assessment'. I am also not convinced of your argument that "putting together different lines of evidence and filling holes in research, got the IPCC involved in conducting new research". I don't see how the emphasis on consensus or quantifying uncertainty counts as conducting new research or filling holes in evidence. Further, you do not mention that the reports undergo a peer review process, similar to other scientific work. 

 Some other minor notes:

 In the introduction you state that “Part of its job is to provide policymakers with the necessary information for making the best decisions in addressing this problem.”.  While in the abstract you state that “main objective is to supply policymakers with policy-relevant recent scientific information about climate change.”. Which is it? The main job or part of its job? If the latter, what is the other part? 

 You miss the fact that the IPCC’s assessment style may have changed, particularly from AR1 to AR6. You treat the work of all IPCC Working Groups as the same, yet most of your examples are from Working Group 1.

If any, are all the source data and materials underlying the results available?

No source data required

Is the work original in terms of material and argument?

Yes

Is the work clearly and cogently presented?

Partly

Is the argument persuasive and supported by evidence?

No

Does the research article contribute to the cultural, historical, social understanding of the field?

Yes

Does it sufficiently engage with relevant methodologies and secondary literature on the topic?

No

Reviewer Expertise:

Science-policy interface, IPCC, Climate change mitigation

I confirm that I have read this submission and believe that I have an appropriate level of expertise to state that I do not consider it to be of an acceptable scientific standard, for reasons outlined above.

# Reviewer report: Reviewer response for version 1

**DOI:** 10.21956/openreseurope.19558.r44607

**Published:** 2024-10-21

**Authors:** David Francisco Bustos Usta

**Affiliations:** 1 Universidad de Concepcion, Concepción, Bío Bío Region, Chile

The article offers an in-depth analysis of the assessment process used by IPCC. The main goal is to provide insights into the specific type of assessment the IPCC performs and the factors that shaped it.

 The manuscript starts by exploring the origins of the IPCC and the reasons for its creation. Therefore emphasizes on climate-scientific-specific obstacles that the IPCC faced, such as the integration of interdisciplinary knowledge, the need for consensus-building, uncertainty quantification, and addressing gaps in knowledge. Finally, the author explores some controversies surrounding the IPCC's assessments, which arise from the specific ways it addresses these scientific challenges.

 There are some points that could improve this manuscript:

 1. The article could benefit from a clearer, more structured definition of what "assessment style" means, particularly in distinguishing the IPCC’s approach from other expert organizations. For instance, a table or framework contrasting the IPCC's assessment style with other global organizations would enhance clarity.

 2. While the obstacles are well-explained, the article could delve deeper into how these scientific challenges have evolved over time. For example, the discussion could highlight how the handling of uncertainty or interdisciplinary integration has improved (or not) across IPCC reports, especially in the transition from AR5 to AR6.

 3. Regarding Section 5.2. Can the authors offer more specific suggestions on how the IPCC could improve its communication of uncertainty?, that would be great

 4. More concrete examples of how the IPCC has balanced consensus-building with the need to represent scientific diversity would strengthen the argument.

If any, are all the source data and materials underlying the results available?

No source data required

Is the work original in terms of material and argument?

Yes

Is the work clearly and cogently presented?

Partly

Is the argument persuasive and supported by evidence?

Partly

Does the research article contribute to the cultural, historical, social understanding of the field?

Yes

Does it sufficiently engage with relevant methodologies and secondary literature on the topic?

Yes

Reviewer Expertise:

Climate Change, Climate models, Artificial Intelligence and Oceanography

I confirm that I have read this submission and believe that I have an appropriate level of expertise to confirm that it is of an acceptable scientific standard, however I have significant reservations, as outlined above.

# Reviewer report: Reviewer response for version 1

**DOI:** 10.21956/openreseurope.19558.r43359

**Published:** 2024-10-16

**Authors:** Hannah Hughes

**Affiliations:** 1 Aberystwyth University, Aberystwyth, Wales, UK

This article sets out to address really important questions about the IPCC and its assessment style: asking what exactly is it that the Panel is doing, why is it doing it in that particular way and why what it does was/is perceived by some as controversial? I am reviewing the article you have written to address these questions from the standpoint of someone who has attempted to address very similar questions and provided a detailed account of the organisation and its practice for producing assessments of climate change (Hughes 2015; Hughes and Paterson 2017; Hughes 2023; Hughes 2024). You do not have to engage with all of these, but as with the literature identified below, doing so may help you to further articulate and strengthen the unique contribution that you are attempting to make in your article and to situate this in relation to existing scholarship.

 From the outset of your article, you suggest that there are two strategies for tackling these questions: 1) to focus on social and political factors in the organisations establishment and 2) concentrate on the climate-scientific-specific obstacles that the Panel had to overcome in trying to do what it has been mandated by the UN General Assembly (UNGA). You indicate that by looking at the solutions the panel found for dealing with these obstacles we can essentially ‘know’ its assessment style and shed light on the controversies this has created. You identify the climate-scientific-specific obstacles as: 1) the need to address consensus-issues, 2) the inherent uncertainty of climate-scientific results, 3) the interdisciplinary nature of climate research, and 4) the gaps in knowledge relevant to climate change. You conclude the Panel's response to these obstacles acted as both the main constraining factor on its assessment style and the principal source of controversies associated with it over social and political factors (and presumably accounts of this assessment style). I find this conclusion a bit troubling however, for the following reasons:

 First, you don’t tell us what the strategy of exploring the IPCC/assessment style through social and political factors has taught us. This literature is not reviewed and you do not indicate where our understanding or knowledge is lacking as a result of following this strategy. Without knowing what the present gap in understanding is it is hard to determine what you contribute by taking this alternative strategy and focusing on the climate-scientific-specific obstacles.

 Second, is it a new approach? Many scholars interested in the IPCC have tried to understand how the IPCC has addressed the climate-scientific-specific obstacles that you provide an account of, but you don’t review these and at one point even indicate there isn’t the space to provide this review (I am thinking here particularly of some of the early STS literature on the IPCC and uncertainty e.g. work by Brian Wynne and Simon Shackley ). There may not be the space for a thorough review of this scholarship, but you can provide an account of what we know and what you’re showing us differently with reference to these.

 Thirdly, what about those scholars that have considered both and built both factors into their frameworks and analyses? Many scholars from the outset would indicate that addressing the question you set out with cannot be answered with an either or approach. They have adopted different concepts and methods to capture this, revealing how the scientific order (conventions, practices and forms of authority) informed leaders approach to fulfilling the practical task of producing the assessment combined with an account of the social and political forces structuring the emergence of the organisation and its assessment practice (Hughes 2024; but also accounts informed by metaphor of co-production e.g. Miller 2004). Through close up examination of particular shifts across assessment cycles, in relation to WAIS for example, this scholarship illuminates the interplay between science and cultural forces in the assessment of a specific topic (O’Reilly, Oreskes and Oppenheimer 2012 ).

 I am sure your article has unique insights and a contribution to make on how  climate-scientific-specific obstacles are overlooked in how we understand the IPCC’s assessment style (although, I don’t think that you can know the organisation or its assessment practice through these alone). However, to demonstrate the importance of taking this focused approach you need to develop more nuance in your argument through engaging more carefully and thoroughly with what we know and how we know what we do about the IPCC today, and then showing us what we could understand differently and more clearly through your research strategy.

 Finally, your use of ‘panel’ and IPCC may need more consideration to bring greater clarity to exactly who in the organisation or which part of the organisation you are referring to. This really becomes noticeable as the article progresses and you begin to articulate and illustrate the central elements e.g.: *In trying to overcome the first obstacle (i.e. the hidden errors in the research), the IPCC had to act as an epistemic authority in charge of providing quality control for all the research output relevant to the question of climate change. *But who are you referring to as the IPCC here? I notice you’re not using panel anymore, e.g. member governments. Do you mean the bureau. They are likely to be discussing it and dealing with it in a very practical way at the outset and during an assessment cycle, and it’s very likely that the co-chairs and the technical support units will develop papers and guidance notes to inform authors on how to tackle the specific issues you raise (grey literature, cut off date for publication, guidance on treatment of uncertainty), as well as checking the draft chapters and harmonising the final report to ensure these are adhered to.

 Below I Iist some of the articles and books that I have written to try to address similar questions about what the IPCC is? What it does? And how it came to be this way? Importantly, I was driven by a different underlying question, namely what constitutes symbolic power/authority to determine the meaning of this collective problem? However, when I set out to address this question, learning with/from existing IPCC literature and then progressed to the interview stage, it quickly became apparent that this scholarship didn’t yet provide a detailed account of what the IPCC is and does, which became an important motivation for my research and the outputs below.

 Hughes, H. ‘2024 Ref 1

 Hughes, H. 2024. Ref 2

 Hughes, H. 2015  Ref 3

 Hughes, H. 2017 Ref 4 

 Miller, C. (2004) Ref 5

 O’Reilly, J., Oreskes, N., & Oppenheimer, M. (2012) Ref 6

 Shackley, Simon, and B. Wynne. 1995. Ref 7

 Shackley, S, and B Wynne. (1996) Ref 8

 Shackley, S., P. Young, S. Parkinson, and B. Wynne. (1998) Ref 9

If any, are all the source data and materials underlying the results available?

Partly

Is the work original in terms of material and argument?

Partly

Is the work clearly and cogently presented?

Yes

Is the argument persuasive and supported by evidence?

Partly

Does the research article contribute to the cultural, historical, social understanding of the field?

Partly

Does it sufficiently engage with relevant methodologies and secondary literature on the topic?

Partly

Reviewer Expertise:

NA

I confirm that I have read this submission and believe that I have an appropriate level of expertise to confirm that it is of an acceptable scientific standard, however I have significant reservations, as outlined above.

## References

[1] : Actors, activities, and forms of authority in the IPCC. *Review of International Studies* .2024;50(2) : 10.1017/S0260210523000207 333-353 10.1017/S0260210523000207

[2] : The IPCC and the Politics of Writing Climate Change.2024; 10.1017/9781009341554 10.1017/9781009341554

[3] : Bourdieu and the IPCC’s Symbolic Power. *Global Environmental Politics* .2015;15(4) : 10.1162/GLEP_a_00323 85-104 10.1162/GLEP_a_00323

[4] : Narrowing the Climate Field: The Symbolic Power of Authors in the IPCC's Assessment of Mitigation. *Review of Policy Research* .2017;34(6) : 10.1111/ropr.12255 744-766 10.1111/ropr.12255

[5] States of Knowledge.2004; 10.4324/9780203413845 10.4324/9780203413845

[6] : The rapid disintegration of projections: the West Antarctic Ice Sheet and the intergovernmental panel on climate change. *Soc Stud Sci* .2012;42(5) : 10.1177/0306312712448130 709-31 10.1177/0306312712448130 23189611

[7] : Global Climate Change: The Mutual Construction of an Emergent Science-Policy Domain. *https://www.research.ed.ac.uk/en/publications/global-climate-change-the-mutual-construction-of-an-emergent-scie* .1995;

[8] : Representing Uncertainty in Global Climate Change Science and Policy: Boundary-Ordering Devices and Authority. *Science, Technology, & Human Values* .1996;21(3) : 10.1177/016224399602100302 275-302 10.1177/016224399602100302

[9] : Uncertainty, complexity and concepts of good science in climate change modelling: Are GCMs the best tools?. *https://www.research.ed.ac.uk/en/publications/uncertainty-complexity-and-concepts-of-good-science-in-climate-ch* .1998;

# Reviewer report: Reviewer response for version 1

**DOI:** 10.21956/openreseurope.19558.r44604

**Published:** 2024-10-14

**Authors:** Kari De Pryck

**Affiliations:** 1 University of Geneva, Geneva, Switzerland

The article explores the type of assessment the IPCC performs. While the question is interesting, the research protocol is, in my view, unsatisfactory. From a social science perspective, the paper lacks a clear theoretical framework, methodology, and analytical protocol.

 First, the paper should be more firmly situated within Science and Technology Studies (STS), as many of the articles cited rely on STS frameworks. STS scholars, by focusing on practices, have demonstrated that assessment-making involves expert judgment and discernment. Oppenheimer et al.'s definition of assessment as “a kind of discernment, where experts gather and evaluate evidence and attempt to discriminate among diverse, competing, and sometimes conflicting claims” is particularly relevant. From an STS perspective, there isn't a single way to conduct an assessment, but multiple approaches, shaped by cultural and institutional factors – see in particular Ref 1.

 The following two articles have also shown how characterizing uncertainty involves cultural and social factors.

 Mach, K.J. et al. (2017) Ref 2 Swart, R. et al. (2009) Ref 3

 Second, it is unclear how the author distinguishes between 'climate-scientific-specific' and social and political factors, as well as the rationale for making such a distinction. In my view, the analysis underscores the importance of social, cultural, and political factors in understanding the assessment process within the IPCC. Questions such as how rules and procedures shape the assessment, the definition of the mandate, and the AR4 errors are crucial. For instance, the errors regarding the Himalayan glaciers are tied to the use of grey literature in the assessment, the role of review editors in identifying errors (external reviewers had flagged them), and the Bureau's delayed acknowledgment of the mistakes—thus highlighting social and institutional factors. See the following articles

 O’Reilly, J. (2015) Ref 4” in J. Barnes and M.R. Dove Ref 5

 Beck, S. (2012) Ref 6

 De Pryck, K. (2021) Ref 7

 Third, the methodology requires clarification and enhancement. First, I recommend that the author use databases such as Scopus or Web of Science to identify a comprehensive set of articles for the review. Second, while the example of the ECS is interesting, the analysis should not be solely based on report assessments. I encourage the author to conduct interviews with the IPCC authors responsible for assessing ECS in AR6 to gain insights into how they arrived at the final figures, as well as to analyse the review comments.

If any, are all the source data and materials underlying the results available?

No source data required

Is the work original in terms of material and argument?

Partly

Is the work clearly and cogently presented?

Partly

Is the argument persuasive and supported by evidence?

Partly

Does the research article contribute to the cultural, historical, social understanding of the field?

Partly

Does it sufficiently engage with relevant methodologies and secondary literature on the topic?

No

Reviewer Expertise:

IPCC, social studies of science

I confirm that I have read this submission and believe that I have an appropriate level of expertise to state that I do not consider it to be of an acceptable scientific standard, for reasons outlined above.

## References

[1] : The rapid disintegration of projections: the West Antarctic Ice Sheet and the intergovernmental panel on climate change. *Soc Stud Sci* .2012;42(5) : 10.1177/0306312712448130 709-31 10.1177/0306312712448130 23189611

[2] : Unleashing expert judgment in assessment. *Global Environmental Change* .2017;44: 10.1016/j.gloenvcha.2017.02.005 1-14 10.1016/j.gloenvcha.2017.02.005

[3] : Agreeing to disagree: uncertainty management in assessing climate change, impacts and responses by the IPCC. *Climatic Change* .2009;92(1-2) : 10.1007/s10584-008-9444-7 1-29 10.1007/s10584-008-9444-7

[4] : 4. Glacial Dramas: Typos, Projections, and Peer Review in the Fourth Assessment of the Intergovernmental Panel on Climate Change.2020; 10.12987/9780300213577-006 107-126 10.12987/9780300213577-006

[5] : Climate Cultures Anthropological Perspectives on Climate Change. *https://yalebooks.yale.edu/book/9780300198812/climate-cultures* .2015;

[6] : Between Tribalism and Trust: The IPCC Under the "Public Microscope". *Nature and Culture* .2012;7(2) : 10.3167/nc.2012.070203 151-173 10.3167/nc.2012.070203

[7] : Controversial Practices: Tracing the Proceduralization of the IPCC in Time and Space. *Global Policy* .2021;12(S7) : 10.1111/1758-5899.12910 80-89 10.1111/1758-5899.12910

# Reviewer report: Reviewer response for version 1

**DOI:** 10.21956/openreseurope.19558.r44601

**Published:** 2024-10-01

**Authors:** Tomàs Molina

**Affiliations:** 1 University of Barcelona, Barcelona, Catalonia, Spain

This piece reads more like an essay or commentary on the issue of the IPCC assessments rather than a traditional research article. While the subject is highly relevant, and the discussion is well-executed and of great interest, the text can be difficult to follow at times. The treatment of footnotes and references is somewhat confusing due to multiple approaches to consultation

 Overall, I believe the article is ready for approval with minor revisions.

 Suggestions for improvement:

- Section 2: It would be helpful to mention the First World Climate Conference (1979), which led to the creation of UNEP and, eventually, the IPCC.
- Section 3: The reference to "(p.57)" is unclear, particularly concerning which book or paper is being cited. If it pertains to Reference 1, the phrase “family resemblance” only appears once, on the first page. I haven't checked all page references, but they seem somewhat confusing.
- Section 3.1: The acronym "UNGA" has not been defined.

Main Question for the Author:

 The research question, as stated in the introduction and title—“What follows is the result of an epistemological effort to identify those climate-scientific-specific factors that were at least very important, if not crucial, in shaping the IPCC’s assessment style”—does not fully align with the conclusions. The conclusion states: “The main objective of this paper was to explore a different strategy for accounting for an important aspect of the IPCC’s activity: the type of assessment that it performs in order to obtain policy-relevant scientific information about climate change.”

 After such an in-depth discussion that often circles back, it would be beneficial to expand the conclusions by summarizing some key points from the discussion. This would help in identifying the "climate-scientific-specific factors" that were instrumental in shaping the IPCC’s assessment style. As it stands, the conclusions feel too brief and vague, especially considering the length of the preceding discussion.

If any, are all the source data and materials underlying the results available?

No source data required

Is the work original in terms of material and argument?

Yes

Is the work clearly and cogently presented?

Yes

Is the argument persuasive and supported by evidence?

Yes

Does the research article contribute to the cultural, historical, social understanding of the field?

Yes

Does it sufficiently engage with relevant methodologies and secondary literature on the topic?

Yes

Reviewer Expertise:

Meteorology, Climate Change Communication

I confirm that I have read this submission and believe that I have an appropriate level of expertise to confirm that it is of an acceptable scientific standard.

## References

[1] : The future of global environmental assessments: Making a case for fundamental change. *The Anthropocene Review* .2021;8(1) : 10.1177/2053019620971664 56-82 10.1177/2053019620971664
